# Plant Resources, ^13^C-NMR Spectral Characteristic and Pharmacological Activities of Dammarane-Type Triterpenoids

**DOI:** 10.3390/molecules21081047

**Published:** 2016-08-12

**Authors:** Jingya Ruan, Chang Zheng, Lu Qu, Yanxia Liu, Lifeng Han, Haiyang Yu, Yi Zhang, Tao Wang

**Affiliations:** 1 Tianjin State Key Laboratory of Modern Chinese Medicine, 312 Anshanxi Road, Nankai District, Tianjin 300193, China; ruanjy19930919@163.com (J.R.); qululuhan88@163.com (L.Q.); liuyanxia210@163.com (Y.L.); 2 Tianjin Key Laboratory of TCM Chemistry and Analysis, Institute of Traditional Chinese Medicine, Tianjin University of Traditional Chinese Medicine, 312 Anshan Road, Nankai District, Tianjin 300193, China; 18702270347@163.com (C.Z.); hanlifeng_1@sohu.com (L.H.); yuhaiyang19830116@hotmail.com (H.Y.)

**Keywords:** Dammarane-type triterpenoids, plant resources, NMR spectral characteristics, biological activities

## Abstract

Dammarane-type triterpenoids (DTT) widely distribute in various medicinal plants. They have generated a great amount of interest in the field of new drug research and development. Generally, DTT are the main bioactive ingredients abundant in Araliaceae plants, such as *Panax ginseng*, *P. japonicas*, *P. notoginseng*, and *P. quinquefolium*. Aside from Araliaceae, DTT also distribute in other families, including Betulaceae, Cucurbitaceae, Meliaceae, Rhamnaceae, and Scrophulariaceae. Until now, about 136 species belonging to 46 families have been reported to contain DTT. In this article, the genus classifications of plant sources of the botanicals that contain DTT are reviewed, with particular focus on the NMR spectral features and pharmacological activities based on literature reports, which may be benefit for the development of new drugs or food additives.

## 1. Introduction

**Figure 1:**
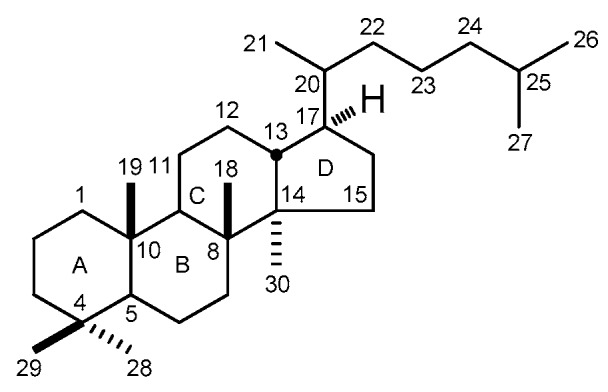
The basic skeleton of dammarane-type triterpenoids.

Dammarane-type triterpenoids (DTT) belong to tetracyclic ring triterpenoids. Their structural characteristic is with 5α-H, C_8_β-CH_3_, 9α-H, C_10_β-CH_3_, C_13_β-H, C_14_α-CH_3_, C_17_β-side chain, and 20*R* or *S* configuration (Figure 1). Usually, C-3, -6, -7, -12, -20, -23, -24, or -25 are replaced by hydroxyl group; C-3, -6, or -20 are substituted by saccharide groups; and olefinic bond are formatted between C-5 and -6, C-20 and -21, C-20 and -22, C-22 and -23, C-24 and -25 or C-25 and -26. Moreover, cyclization generally displays at C_17_-side chain. Specifically, a five-membered ring with epoxy bond is usually formed between C-20 and C-24, a five-membered lactone ring usually appears between C-21 and C-23, and a six-membered ring with epoxy bond displays between C-20 and C-25 for DTT. They are usually classified into protopanaxdiol (PPD) and protopanaxtriol (PPT, with 6-OH) groups based on their aglycone moieties.

As one of the main secondary metabolites of a number of Traditional Chinese Medicines (TCM), DTT have gained more and more attention around the world owing to their remarkable biological activities [1], and display specific plant distribution.

In order to complete and enrich the resource investigation of DTT, we summarize the literatures (1965–2016) describing this type of triterpenoids, which were extracted from various botanicals. Thus, 136 species, 79 genera, and 46 families containing DTT are summarized to reveal their plant sources.

As is known, pharmacodynamic substance research is based on structural determination, among various structural analysis methods such as ultraviolet, infrared, optical rotation, circular dichroism, nuclear magnetic resonance (NMR), and Mass spectral analysis. NMR plays an important role in structural identification. Here, the characteristics of ^1^H- and ^13^C-NMR spectra for DTT together with the ^13^C-NMR chemical shift changes caused by various substituent groups for DTT are summarized. The work may be helpful to discriminate DTT rapidly and conveniently.

Furthermore, in pharmacological research, DTT, as well as their derivatives, showed various bioactivities such as anti-tumor, anti-inflammatory, immunostimulatory, neuronal cell proliferatory, anti-aging, anti-bacterial, anti-diabetes, and anti-osteoporosis abilities. Among the multiple DTT, ginsenoside Rg_3_ as the first anti-cancer monomer isolated from TCM, has been applied as a kind of auxiliary anti-cancer drug to increase efficacy and release of the chemotherapy-induced symptoms, and has been proven to be effective and safe [2,3]. Why does ginsenoside Rg_3_, a relatively rare DTT obtained from *P. ginseng*, exhibit excellent biological activity? Do other DTT perform similar ability? The explanations of their structure-activity relationships (SARs) summarized in the following might be helpful to answer these questions.

## 2. Plant Resources of DTT

In order to complete and enrich the resource investigation of DTT, we summarize the literatures (1965–2016) describing this type of triterpenoids, extracted from various botanicals. In Table 1, 136 species, from Araliaceae, Cucurbitaceae, Rhamnaceae, and Meliaceae families, together with 42 others are summarized [4,5,6,7,8,9,10,11,12,13,14,15,16,17,18,19,20,21,22,23,24,25,26,27,28,29,30,31,32,33,34,35,36,37,38,39,40,41,42,43,44,45,46,47,48,49,50,51,52,53,54,55,56,57,58,59,60,61,62,63,64,65,66,67,68,69,70,71,72,73,74,75,76,77,78,79,80,81,82,83,84,85,86,87,88,89,90,91,92,93,94,95,96,97,98,99,100,101,102,103,104,105,106,107,108,109,110,111,112,113,114,115,116,117,118,119,120,121,122,123,124,125,126,127,128,129,130,131,132].

## 3. NMR Spectral Characteristic of DTT

Meanwhile, more than 760 kinds of DTT were reported from 1965 to 2016. Summarizing the DTT NMR data, we know that the characteristics of ^1^H-NMR spectra for dammarane-type sapogenin are always seven or eight singlet that belong to methyl signals in the high field δ 0.6~1.5. The chemical shift values of olefinic protons usually locate in δ 4.3~6.0, and the proton signals of oxygen carbon may appear in δ 4.0~5.5. For the ^13^C-NMR spectra, the chemical shift values are usually divided in 3~4 ranges: δ 8.0~60.0 (methyl, methene, methine, and quaternary carbon; angular methyl generally located in 8~35), δ 60.0~90.0 (oxygen-methine and quaternary carbon), δ 109.0~160.0 (olefinic carbon), and 170.0~220.0 (carbonyl carbons).

Besides these NMR techniques for structure identification of natural products, application of chemical shift rules summarized from reports on similar type compounds will be useful for structure determination.

Here, the aglycone parts’ NMR data of 33 representative common DTT were chosen to summarize the NMR chemical shift rules caused by varieties of substituent groups such as hydroxyl, carbonyl, olefinic bond, glycosyl, and cyclization. The work may be helpful to discriminate DTT rapidly and conveniently. As the ^13^C-NMR occupy more crucial positions than their ^1^H-NMR, the examples listed below were primarily elucidated by their carbon chemical shift values (Table 2).

### 3.1. Hydroxyl

Hydroxyls usually occur at C-3, -6, -12 and/or -20 positions, however, the chemical shift values caused by hydroxyl substitution are different from each other (δ_C-3_ ~78, δ_C-6_ 66~68, δ_C-12_ 70~72, δ_C-20_ ~74, in C_5_D_5_N or CDCl_3_, Table 2).

**Figure 2:**
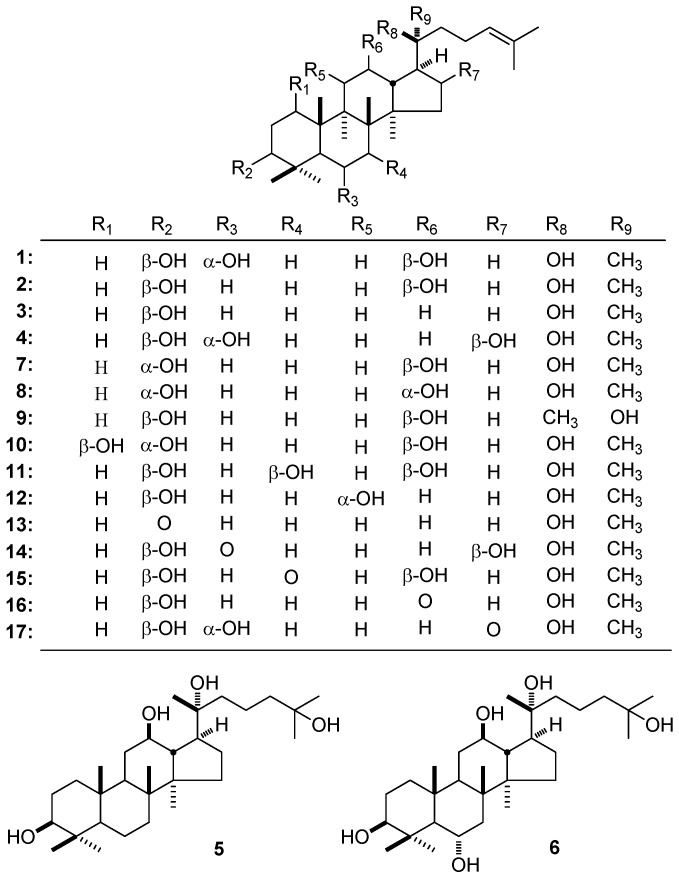
The structure of compounds **1**–**17**.

Among them, 6-OH is always the iconic difference between PPD and PPT (Figure 2). When there is a hydroxyl substituted at C-6, for example, 20*S*-PPT (**1**) vs. 20*S*-PPD (**2**), the ^13^C-NMR signals for the carbons except C-9 on B ring shift downfield. Meanwhile, those of C-18 and -19, which are adjacent to B ring, change the same way. On the other hand, because of the spatial relationship between C_6_-OH and 28-CH_3_, downfield displacement of C-28 exerts as well [133,134]. Meanwhile, comparing the NMR data of 20*S*-dammarenediol 1a (**3**) [135] and horipenoid E (**4**) [59], 20*R*-dammarane-3β,12β,20,25-tetrol (20*R*-25-OH PPD) (**5**), and 20*R*-dammarane-3β,6α,12β,20,25-tetrol (20*R*-25-OH PPT) (**6**) [136], the same hydroxyl substitution effects were observed. Here, the downfield shifts of C-5–7 could be explained by the α- and β-effects of hydroxyl substitution at C-6. In theory, the γ-effect of 6-OH should induce the upfield shifts of C-8 and C-10, the opposite between the fact and theory may be due to electron cloud density reduction around C-8 and C-10 caused by conformational change with the introduction of 6-OH.

Though the hydroxyl substituted at C-3, -12, and -20 positions is commonly in 3β, 12β, and 20*S* configuration, there may be some transformations occasionally. The configuration changes could cause various changes in chemical shift values. For example, the ^13^C-NMR signals for C-1–5 of betulafolienetriol (**7**) [72] [δ 25.4 (C-2), 33.6 (C-1), 37.6 (C-4), 49.5 (C-5), 76.2 (C-3)] with 3α-OH are in upper field than those of 20*S*-PPD (**2**) [134] [δ 27.4 (C-2), 39.0 (C-1), 39.0 (C-4), 56.0 (C-5), 78.8 (C-3)] with 3β-OH (Table 2). However, 3α-OH substitution will make the δ value of C-29 shift downfield (**2**: δ 15.5; **7**: δ 22.1). While when 12-OH performs α-configuration, the influence seems wider, the signals for carbons on C and D rings shift upfield in different levels [(3α,12α)-dammar-24-ene-3,12,20-triol (**8**) [134]: δ 24.1 (C-16), 29.0 (C-11), 45.3 (C-9 and C-13), 46.9 (C-17), 48.8 (C-14), 68.4 (C-12) vs. **7** [72]: δ 26.5 (C-16), 30.9 (C-11), 47.7 (C-13), 49.9 (C-9), 51.7 (C-14), 53.5 (C-17), 71.0 (C-12)]. That of C-22 changes slightly (**8**: δ 36.3; **7**: δ 34.7) because of the spatial proximity between 12-OH and C-22. Moreover, the configuration of 20-OH will mainly affect the chemical shift of C-17, -21, and -22 [20*R*-PPD (**9**) [134]: δ 21.8 (C-21), 42.3 (C-22), 49.9 (C-17) vs. 20*S*-PPD (**2**) [134]: δ 26.8 (C-21), 34.8 (C-22), 53.6 (C-17)]. According to the above-summarized rules, we can clarify the configuration of DTT rapidly (Table 2) (Figure 2).

Besides the common hydroxyl substituted position mentioned above, there are also some special examples as following: (1) When C-1 is replaced by a hydroxyl group, the NMR signals of C-1–3, 7–12 shift to lower field, but those of C-5 and -19 shift to upper field [probosciderol B (**10**) [72] vs. **7** [72]); (2) 7-OH substitution may not only cause the δ values of C-6–8 shift downfield, but also influence those of C-5, -15, -16, and -18 [7β-hydroxyl 20*S*-protopanaxatriol (**11**) [137]: δ 10.7 (C-18), 27.6 (C-6), 28.5 (C-16), 36.1 (C-15), 46.0 (C-8), 54.2 (C-5), 74.7 (C-7) vs. **2** [133]: δ 16.2 (C-18), 18.7 (C-6), 26.8 (C-16), 31.8 (C-15), 35.2 (C-7), 40.0 (C-8), 56.3 (C-5)]; (3) The effects of the 11-OH are profound, spatial related carbon exert chemical shift values in different degrees [dammar-24-en-3β,11α,20*S*-triol (**12**) [138]: δ 39.6 (C-10), 40.8 (C-12), 41.1 (C-13), 41.7 (C-1), 41.8 (C-22), 50.3 (C-17), 56.1 (C-9), 70.5 (C-11) vs. **2** [133]: δ 32.0 (C-11), 35.8 (C-22), 37.3 (C-10), 39.5 (C-1), 48.5 (C-13), 50.4 (C-9), 54.7 (C-17), 70.9 (C-12)]; (4) The comparison of [20*S*-dammarenediol 1a (**3**) [139] and 20*S*-PPD (**2**) [134], along with (3α,12α)-dammar-24-ene-3,12,20-triol (**8**) [134] and dammar-24-en-3β,11α,20*S*-triol (**12**) [138], indicate that 12-OH substitution may influence the chemical environment around the C-11–14, 16, 17, and 20–22 (Table 2) (Figure 2). The above-mentioned changes could be explained by the α-, β-, and γ-effects and conformational change with the introduction of hydroxyl.

### 3.2. Carbonyl

Carbonyl always derives from the oxidation of hydroxyl. This is why carbonyl usually locates at C-3, -6, -7 and/or -12. When carbonyl appears at the C-3 position, the influence is limited to carbons right next to it as well as the C-29 methyl, all the influenced carbon signals shift downfield in different levels [20*S*-20-hydroxydammar-24-en-3-one (**13**) [119]: δ 21.0 (C-29), 34.1 (C-2), 47.4 (C-4), 218.2 (C-3) vs. 20*S*-dammarenediol 1a (**3**) [135]: δ 15.4 (C-29), 27.4 (C-2), 39.1 (C-4), 78.9 (C-3)]. Though the carbonyl substitution at C-6 is not common, there has still been a rule that almost all the related carbon exerted downfield displacement except C-10 which may be caused by the spatial relationship [horipenoid G (**14**) [59]: δ 16.7 (C-18), 17.6 (C-19), 28.5 (C-28), 38.8 (C-4), 44.5 (C-10), 47.3 (C-8), 53.7 (C-7), 66.3 (C-5), 212.3 (C-6) vs. horipenoid E (**4**) [59]: δ 17.8 (C-18), 18.8 (C-19), 32.4 (C-28), 39.7 (C-10), 40.7 (C-4), 41.9 (C-8), 48.3 (C-7), 62.1 (C-5), 67.9 (C-6)]. Owing to the existence of carbonyl at C-7, the signals of carbons around C-7 shift downfield except C-5 and -14 (which performs upfield shift) [7-oxo-20*S*-protopanaxatriol (**15**) [135] δ 18.8 (C-30), 33.2 (C-15), 37.1 (C-6), 49.9 (C-13), 50.2 (C-14), 54.1 (C-5), 56.0 (C-8), 214.0 (C-7) vs. **2** [133]: δ 17.0 (C-30), 18.7 (C-6), 31.8 (C-15), 35.2 (C-7), 40.0 (C-8), 48.5 (C-13), 51.6 (C-14), 56.3 (C-5)]. Carbonyl can appear at C-12, as what we have summarized above, the change at C-12 may affect the carbon related to it as well as the side chain [12-keto-20*S*-protopanaxadiol (3β,20*S*-dihydroxydammar-24-en-12-one) (**16**) [139]: δ 37.8 (C-22), 39.1 (C-11), 46.1 (C-17), 53.4 (C-9), 54.7 (C-14), 56.2 (C-13), 214.1 (C-12) vs. **2** [134]: δ 31.2 (C-11), 34.8 (C-22), 47.7 (C-13), 50.2 (C-9), 51.6 (C-14), 53.6 (C-17), 70.8 (C-12)]. An unusual carbonyl substitution appears at C-16 of PPT like horipenoid H (**17**) [59], as a result, the signals of carbons right next to it move downfield, while the C-14 and C-22 shift upfield [**17** [59]: δ 41.3 (C-22), 45.8 (C-14), 50.5 (C-15), 58.5 (C-17), 220.6 (C-16) vs. **4** [59]: δ 44.3 (C-22), 44.6 (C-15), 48.3 (C-14), 52.2 (C-17), 74.2 (C-16)].

### 3.3. Cyclization

Moreover, cyclization generally displays at C_17_-side chain. A five-membered ring with epoxy bond is usually formed between C-20 and C-24 for DTT, to maintain the consistency of deuterated solvent, here we make a δ values’ comparison between betulafoliene-oxide-I (20*S*,24*R*-epoxy) (**18**) [140] [δ 27.3 (C-26), 27.6 (C-27), 28.7 (C-23), 32.8 (C-22), 49.9 (C-17), 70.1 (C-25), 85.6 (C-24), 86.7 (C-20)] and **2** [133] [δ 17.6 (C-27), 22.9 (C-23), 25.8 (C-26), 35.8 (C-22), 54.7 (C-17), 72.9 (C-20), 126.2 (C-24), 130.6 (C-25)], the characteristic signals belonging to olefinic carbons disappear while the signals of δ 86.7 (C-20) and 85.6 (C-24) occur, which indicate the existence of an epoxy ring. Moreover, the configuration of C-20 and -24 may play an important role in the chemical shifts of carbons around them. For example, betulafoliene-oxide-II (**19**) [140] with 20*S*,24*S*-epoxy displays δ 29.0 and 88.3 for C-27 and -24, respectively. While those of betulafoliene oxide-I (**18**) [140] with 20*S*,24*R*-epoxy are δ 27.6 (C-27) and 85.6 (C-24). However, the configuration change of C-20 can lead itself to shift downfield, but make the carbons related to it shift upfield (20*R*,24*R*-epoxy-3,12,25-triol-dammarane (**20**) [55]: δ 21.3 (C-21), 25.9 (C-23), 39.1 (C-22); **18** [140]: δ 26.9 (C-21), 32.8 (C-22), 28.7 (C-23)).

Meanwhile, a five-membered lactone ring usually appears between C-21 and C-23. The effect of the lactone ring is similar to that of five-membered ring with epoxy bond, except the obvious downfield movement of C-13, the electron-withdrawing effect of the lactone group make the two olefinic carbons shift contrast ((23*S*)-3β-hydroxydammar-20,24-dien-21-oic acid 21,23-lactone (**21**) [141]: δ 37.5 (C-17), 46.7 (C-13), 78.0 (C-23), 121.9 (C-24), 137.8 (C-25), 140.3 (C-20), 145.7 (C-22), 173.9 (C-21) vs. **3** [135]: δ 22.6 (C-23), 24.8 (C-21), 40.5 (C-22), 42.3 (C-13), 49.9 (C-17), 75.4 (C-20), 124.7 (C-24), 131.6 (C-25)).

**Figure 3:**
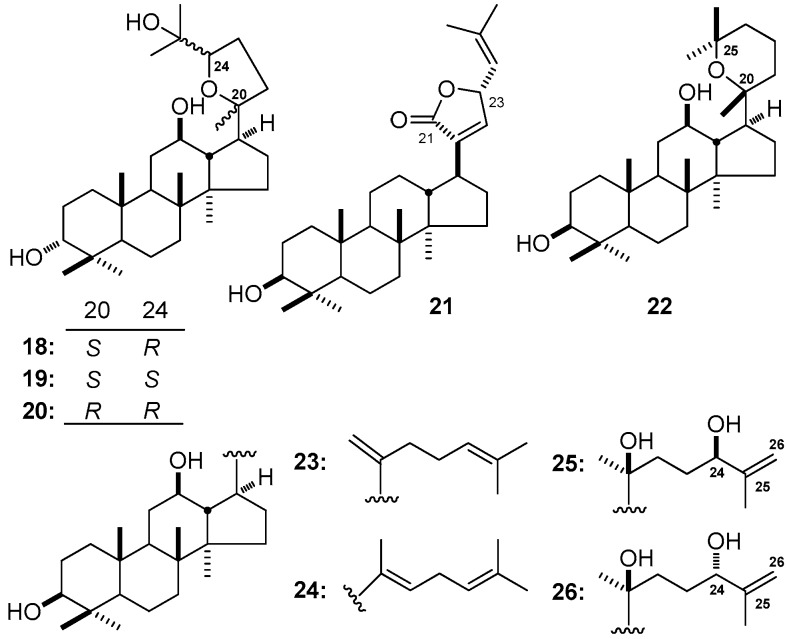
The structure of compounds **18**–**26**.

Furthermore, six-membered ring with epoxy bond displays between C-20 and C-25 is also a special transformation of DTT, the chemical shift changes of it exhibit similar rules like those of five-membered ring substitution (3β,12β-dihydroxy-20*R*,25-epoxydammarane (**22**) [142]: δ 16.3 (C-23), 19.4 (C-21), 27.1 (C-27), 33.0 (C-26), 36.5 (C-24), 73.1 (C-25), 76.7 (C-20) vs. **2** [134]: δ 17.8 (C-27), 22.4 (C-23), 25.8 (C-26), 26.8 (C-21), 74.0 (C-20), 125.2 (C-24), 131.4 (C-25)). Besides the significantly changed positions, the signals of C-13 and C-17 shift downfield slightly as well (Table 2) (Figure 3).

### 3.4. Olefinic Bond

Olefinic bond is one of the most common transformations on the side chain. In this paper, we summarize different combinations of them. The same as the other substitution forms, olefinic bond on the side chain influence not only itself but also the carbons on the D ring in different levels. In general, the substituted carbons appear characterized olefinic carbon signals on ^13^C-NMR, the signals of carbons right next to it shift upfield, while those of the meta- ones exert downfield because of the spatial relationship between them (DHPPD-I (**23**) [143]: δ 27.1 (C-23), 30.8 (C-16), 32.7 (C-22), 33.8 (C-15), 48.2 (C-17), 52.4 (C-13), 72.5 (C-12), 108.1 (C-21), 155.5 (C-20) vs. **2** [133]: δ 22.9 (C-23), 26.8 (C-16), 26.9 (C-21), 31.8 (C-15), 35.8 (C-22), 48.5 (C-13), 54.7 (C-17), 70.9 (C-12), 72.9 (C-20)). When olefinic bond locates between C-20 and C-22, the chemical shift values of C-20 related carbons shift upfield apparently beside C-17 (DHPPD-II (**24**) [143]: δ 13.2 (C-21), 28.7 (C-16), 32.6 (C-15), 50.4 (C-13), 50.9 (C-17)), 123.6 (C-22), 140.1 (C-20) vs. **23**). The changes at C-24 and C-25 mainly affect the signals of carbons on side chain (25,26-en-24*R*-hydroxyl-20*S*-protopanaxadiol (**25**) [144]: δ 30.8 (C-23), 32.1 (C-22), 76.4 (C-24), 110.1 (C-26), 150.2 (C-25) vs. **2** [133]: δ 22.9 (C-23), 25.8 (C-26), 35.8 (C-22), 126.2 (C-24), 130.6 (C-25). Moreover, the different configuration at C-24 may make no sense on the chemical shift, for example, there is nearly no obvious differences between 25,26-en-24*S*-hydroxyl-20*S*)-protopanaxadiol (**26**) [144] and **25** (Table 2) (Figure 3).

### 3.5. Glycosyl

**Figure 4:**
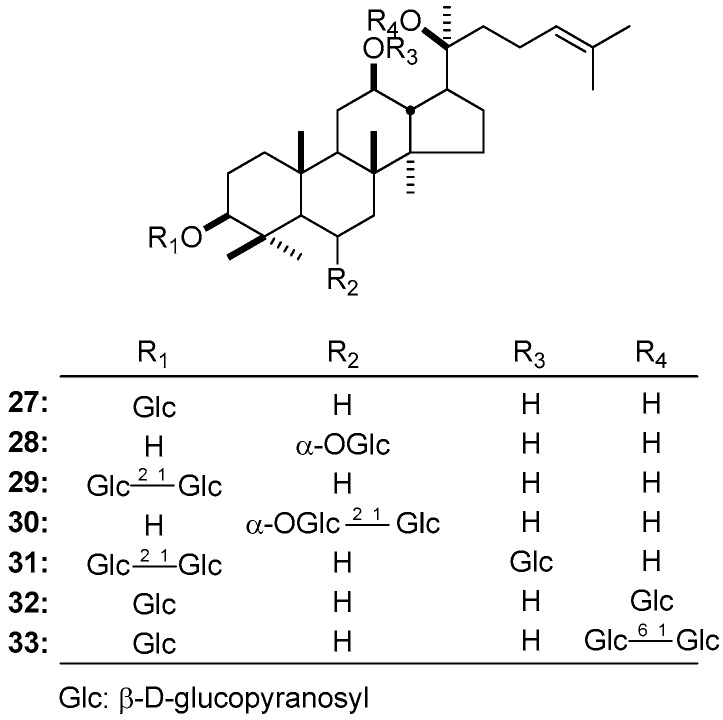
The structure of compounds **27**–**33**.

The hydroxyls of triterpene sapogenin are generally replaced by monosaccharide or polysaccharide, the glycosidation shifts induced by them are at the range of 8~10. When glycosidation displays in C-3, the change of chemical shift values almost exclusively performance in C-2 and -3 [20*S*-ginsenoside Rh_2_ (**27**) [133]: δ 26.8 (C-2), 88.9 (C-3) vs. **2** [133]: δ 28.2 (C-2), 77.9 (C-3)]. The C_6_-glycoside is similar to C_3_-glycoside, the signal of C-6 shifts downfield while C-7 displaces reversely [20*S*-ginsenoside Rh_1_ (**28**) [145]: δ 45.2 (C-7), 80.1 (C-6) vs. **1** [133]: δ 47.4 (C-7), 67.6 (C-6)]. Moreover, the difference of glycosyl substitution would not cause any obvious chemical shift [20*S*-ginsenoside Rg_3_ (**29**) [133] vs. **27** [133]; **28** vs. 20*S*-ginsenoside Rf (**30**) [145]. For the reason that the glycosyl replaced C-12 mainly occurs in the constituents from *P. japonicas* collected in Kumamoto and Miyazaki prefectures, thus we give an example [chikusetsusaponin FK_7_ (**31**) [146]: δ 27.9 (C-11), 46.8 (C-13), 78.5 (C-12) vs. **29** [133]: δ 32.0 (C-11), 48.6 (C-13), 71.0 (C-12)]. Glycosidation at C-20 is also a common situation; different from the previous ones, the changes caused by C_20_-glycoside are not limited to itself and connected ones, it also affects the carbons spatially adjacent [20*S*-ginsenoside F_2_ (**32**) [133] δ 22.5 (C-21), 30.9 (C-11), 51.7 (C-17), 83.4 (C-20) vs. **27** [133]: δ 27.2 (C-21), 32.3 (C-11), 54.8 (C-17), 73.0 (C-20)]. The nucleus of gypenoside XVII (**33**) [133] is nearly the same as **32**; the theory that glycosidation shift would not change as the amount of the glycosides can be confirmed again (Table 2) (Figure 4).

## 4. Pharmacological Effects of DTT

Many herbal medicines containing DTT as major constituents have been reported for their various biological activities, including inflammation, immunodeficiency, cancer, diabetes, fungal infection, bacterial infection, osteoporosis, and central nervous system dysfunction. In this part, we summarized pharmacological activities and SARs of DTT.

### 4.1. Anti-Tumor Activity

In TCM clinic, some herbal medicines, enriched in DTT were used as complementary and alternative agents in cancer treatment, which are helpful for preventing tumor cell metastasis, relieving side effects of radiotherapy and chemotherapy, and improving clinical cure rate, such as *P. ginseng* [147], *P. notoginseng* [148], *D. binecteriferum* [149], etc. Much literatures reported that DTT showed cytotoxicity in many kinds of cancer cell lines.

**Figure 5:**
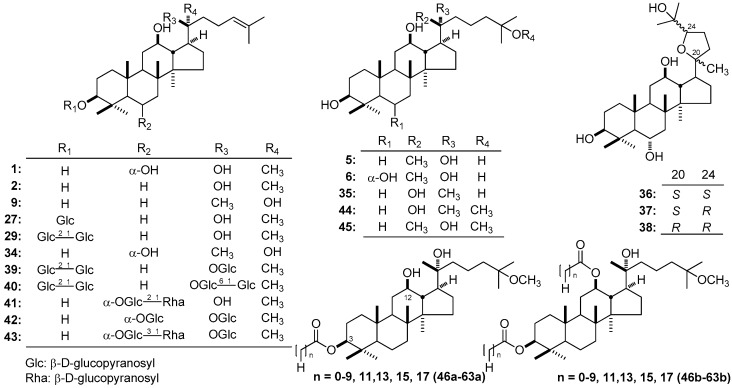
The structure of compounds **1**, **2**, **5**, **6**, **9**, **27**, **29**, **34**–**45, 46a**–**63a**, and **46b**–**63b**.

In vitro experiments have been carried out to analyze the cytotoxicities of DTT obtained from *P*. *ginseng* [150] in three human cancer cell lines, including human leukemia cell line HL-60, human gastric cancer cell line NCI-N87, and human hepatoma cell line HepG2. As a result, 20*S*-PPD (**2**), 20*R*-PPD (**9**), 20*S*-PPT (**1**), 20*R*-PPT (**34**), 20*S*-dammarane-3β,12β,20,25-tetrol (20*S*-25-OH PPD) (**35**), and 20*R*-25-OH PPD (**5**) (Figure 5) showed cytotoxicties against HL-60 cells with IC_50_ at 15.53 ± 0.81, 23.42 ± 0.93, 22.79 ± 3.54, 28.68 ± 6.26, 4.21 ± 0.24, and 11.89 ± 4.04 μM; against NCI-N87 cells with IC_50_ at 50.02 ± 12.21, 56.92 ± 5.11, 53.19 ± 5.77, 65.34 ± 3.62, 60.14 ± 4.70, and 61.76 ± 2.49 μM; and against HepG2 cells with IC_50_ at 45.67 ± 5.22, 69.07 ± 1.49, 43.44 ± 4.87, 58.29 ± 4.15, 6.69 ± 1.86, and 27.12 ± 5.97 μM, respectively. (20*S*,24*S*)-dammar-20,24-epoxy-3β,6α,12β,25-tetraol (**36**), (20*S*,24*R*)-dammar-20,24-epoxy-3β,6α,12β,25-tetraol (**37**), and (20*R*,24*R*)-dammar-20,24-epoxy-3β,6α,12β,25-tetraol (**38**) (Figure 5) did not result in cytotoxicity against these human cancer cell lines. The comparison of the activities between **2** and **9**, **1** and **34**, and **35** and **5** indicated that the configuration at the C-20 would affect their anti-proliferative potency, and the 20*S*-type was stronger than the 20*R*-type. Moreover, their biological effects showed that PPD-type sapogenins may be a little stronger than those of PPT-type sapogenins (**2** vs. **1**, and **9** vs. **34**). On the other hand, the results suggested that whether cyclization at the C-17 side chain (**1** and **34** vs. **36**–**38**), and the presence of 25-hydroxyl group (**2** vs. **35**, and **9** vs. **5**) could play important roles in affecting the anti-proliferative potency.

Four kinds of human cancer cell lines [breast (MCF-7), lung (H838) and prostate (LNCaP (p53 wt) and PC3)] were used to determine the anti-tumor activities of ten dammarane-type terpenoids [20*S*-PPD (**2**), 20*R*-25-OH PPD (**5**), 20*R*-25-OH PPT (**6**), 20*S*-ginsenoside Rh_2_ (**27**), 20*S*-ginsenoside Rg_3_ (**29**), 20*S*-ginsenoside Rd (**39**), 20*S*-ginsenoside Rb_1_ (**40**), 20*S*-ginsenoside Rg_2_ (**41**), 20*S*-ginsenoside Rg_1_ (**42**), and 20*S*-ginsenoside Re (**43**)] with different side-chains (C-22–C-27), different numbers and positions of sugar moieties by Wang et al. [136], and SARs were studied. Anti-proliferative activities order of dammarane-type terpenoids on human cancer cell lines is: PPD-type > PPT-type (25-OH PPD vs. 25-OH PPT) and 25-OH PPD > PPD. Moreover, it indicated that increasing the number of sugar moieties would reduce the anti-proliferative potency. Furthermore, further anti-cancer activity evaluation with thirteen cell lines representing five types of human malignancies (glioma, pancreatic, lung, breast, and prostate) indicated that **2**, **27**, and **5** could inhibit the growth of all cell lines tested, and may be 5–15-fold stronger than those of 20*S*-ginsenoside Rg_3_ (**29**).

It is interesting that though 25-hydroxyl group in PPD-type terpenoids has been found to play important roles in anti-proliferative potency, when it is replaced by methoxyl, the activity is still notable. For example, the IC_50_ values of 20*S*-25-methoxyl-dammarane-3β,12β,20-triol (25*S*-OCH_3_-PPD) (**44**) for most cell lines were in the lower μM order, which is 5–15-fold greater than 20*S*-PPD (**2**) and 10–100-fold higher than compound **29** [151].

Moreover, the importance of hydroxyl substitutions at C-3 and C-12 for anti-proliferative activity of DTT have been evaluated by comparing the cytotoxic activities of 20*R*-25-methoxyl-dammarane-3β,12β,20-triol (25*R*-OCH_3_ PPD) (**45**) and its analogs substituted at the C-3 or C-3 and C-12 positions with fatty acid groups (**46a**–**63a**, and **46b**–**63b**, Figure 5) in four different human tumor cell lines (A549, Hela, HT-29 and MCF-7) and a normal cell line (IOSE144) [152]. Consequently, compounds **45**, **46a**–**63a**, and **46b**–**63b** showed anti-proliferative activities against all tumor cell lines with low toxicities in the normal cell line. SARs of the **45** derivatives suggested that the difference in the substituents may affect the anti-proliferative activity of the compounds. The longer the side chain of **45** is, the lower the anti-proliferative activity would be. On the other hand, the data obtained by Liu et al. indicated that C-3 and C-12 might be active sites of dammarane-type sapogenins and the hydroxyl substitutions at C-3 and C-12 would also be crucial.

**Figure 6:**
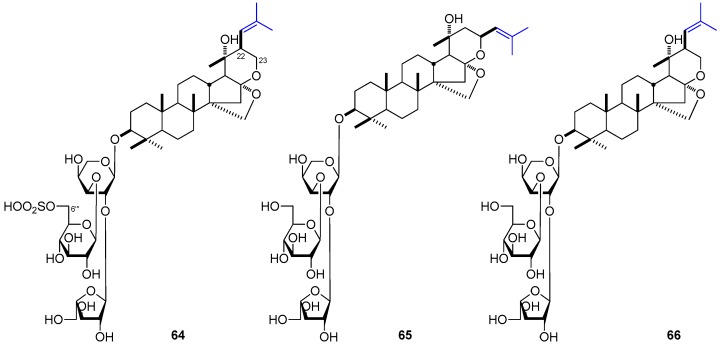
The structure of compounds **64**–**66**.

DTT have been clarified to exhibit significant inhibitory activities to breast cell lines. Bacopasides É (**64**) and VII (**65**) isolated from *B. monniera* [153] could remarkably inhibit human breast cancer cell line MDAMB-231 adhesion, migration and Matrigel invasion in vitro at the concentration of 50 μM. Meanwhile, both **64** and **65** showed strong inhibitory ability in mouse implanted with sarcoma S180 in vivo at 50 μmol/kg. On the other hand, both their in vitro and in vivo activities were obviously stronger than those of their homolog, bacopasaponin C (**66**) (IC_50_: 12.3, 14.3, and 34.9 μM for **64**, **65**, and **66**, respectively). Results revealed that the substitute positions of isobutenyl may play an important role in anti-tumor potency by comparing the activities of **65** and **66**. Besides, the activity would be enhanced by the substitution of sulfonyl at 6′′′ (Figure 6).

**Figure 7:**
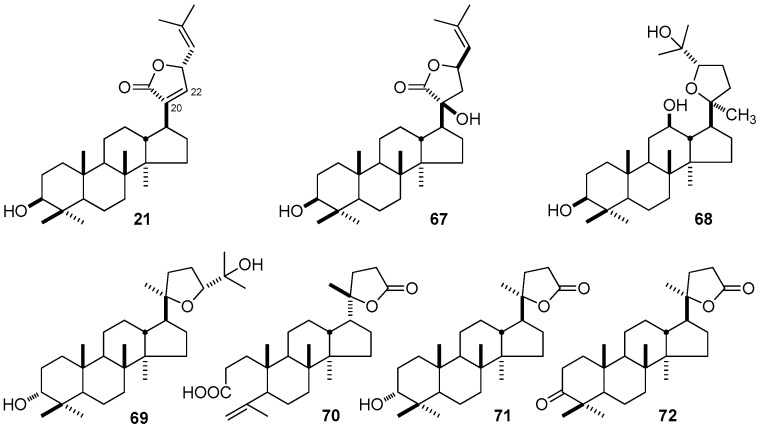
The structure of compounds **21**, **67**–**72**.

Moreover, the cytotoxicity against the human breast cancer cells MDA-MB-435 of three similar DTT with furan ring in their side-chain, (23*S*)-3β-hydroxydammar-20,24-dien-21-oic acid 21,23-lactone (**21**), (20*S*,23*R*)-3β,20β-dihydroxydamma-24-dien-21-oic acid 21,23-lactone (**67**) and (20*S*,24*S*)-20,24-epoxydammarane-3β,12β,25-triol (**68**) were tested. Only compound **21** was found to have significant cytotoxic activity (IC_50_ = 3.9 μg/mL), while **67** and **68** showed no activities, which suggested that the double bond between C-20 and C-22 of the 21,23-lactone moiety might be relatively essential for the cytotoxic activity [141]. According to the experiment carried out by Phongmaykin et al. [84], cabraleadiol (**69**), eichlerialactone (**70**), cabraleahydroxylactone (**71**), and cabralealactone (**72**) (Figure 7) found in *C. penduliflorus* presented weak cytotoxicity against breast cancer line with the IC_50_ values of 17.5, 12.5, 18.0, and 16.9 μg/mL, respectively.

Among the multiple DTT summarized above, ginsenoside Rg_3_, one of characteristic protopanaxadiol ginsenosides of *P. ginseng*, has been studied the most, and has been exploited to be an effective adjuvant therapeutic agent against various cancers. Researchers have demonstrated that it could exhibit protective activities against cervical, prostate, breast, lung, gastric, colorectal, liver, and skin cancer cell lines [154].

The successful clinical applications of ginsenoside Rg_3_ are because it can promote apoptosis, inhibit tumor angiogenesis, inhibit proliferation, invasion and metastasis of tumor cells, impact tumor gene expression signaling, reverse multi-drug resistance, and enhance immunity of patients. Currently, a clinical monomer formulation, “shenyi capsule”, a capsule in combination with chemotherapy, is widely used in a variety of tumors [155].

Although ginsenoside Rg_3_ shows good inhibitory effect of cancer, its poor aqueous solubility and liposolubility are not ideal for clinical applications. Recent studies hves revealed a ginsenoside Rg_3_ bile salt-phosphatidylcholine-based mixed micelle system (BS-PC-MMS) that was carried out using response surface methodology based on a central composite design [156]. Thus, a proper mean for new agents like ginsenoside Rg_3_ has been established to advance the studies of DTT in anti-cancer properties. On the other hand, according to the SARs mentioned above, can we revolve anticancer agent research around ginsenoside Rg_3_, and develop anti-tumor drug with high efficiency and low toxicity?

### 4.2. Anti-Inflammatory Activity

**Figure 8:**
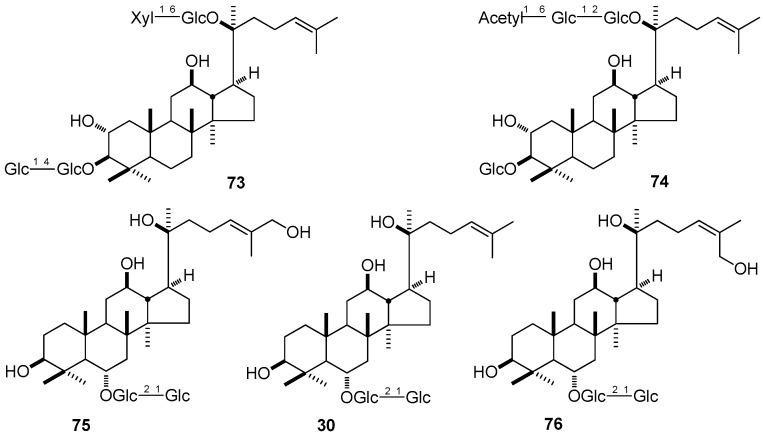
The structure of compounds **30**, **73**–**76**.

Inflammation is considered as the body’s protective response to various chronic diseases, such as, tumor, hypertension and diabetes. The ability that DTT can inhibit inflammation has been predescribed. According to the report [157], when the MTT assay was used to evaluate the cytotoxic effects of 2α,3β,12β,20*S*-tetrahydroxydammar-24-ene-3-*O*-[β-d-glucopyranosyl(1→4)-β-d-glucopyranosyl]-20-*O*-[β-d-xylopyranosyl(1→6)-β-d-glucopyranoside] (**73**) and 2α,3β,12β,20*S*-tetrahydroxydammar-24-ene-3-*O*-β-d-glucopyranosyl-20-*O*-[β-d-6-*O*-acetylglucopyranosyl(1→2)-β-d-glucopyranoside] (**74**) obtained from *G. pentaphyllum*, they showed no cytotoxicities on BEAS-2B cells in either the presence or absence of interleukin-4 (IL-4), but significantly down-regulated IL-4-induced eotaxin production in a concentration-dependent mannar, which indicated that DTT might have potential inflammatory activity, and they may be of benefit to allergic diseases. In addition, three PPT type derivations: ginsenjilinol (**75**), ginsenoside Rf (**30**), and ginsenoside Re_5_ (**76**) isolated from the roots and rhizomes of *P. ginseng* have been proven to exhibit anti-inflammatory activity by inhibiting nitric oxide production by lipopolysaccharide-induced RAW 264.7 [158] (Figure 8).

### 4.3. Immunomodulatory Activity

**Figure 9:**
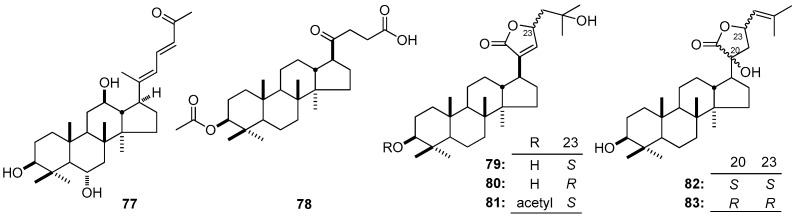
The structure of compounds **77**–**83**.

Immunity is a physiological function of human body. Depending on its feature of identifying “self” and “non-self” components, the antigenic material invading into the body or the cells and tumor cell damage produced by the body itself could be effectively undermined and excluded, which could keep human from being affected by a disease. 27-Demethyl-(*E*,*E*)-20(22),23-dien-3β,6α,12β-trihydroxydammar-25-one (**77**) (Figure 9) has been excavated out from *P. ginseng* by the bioassay-guided assay [159]. As mentioned above, the overproduction of NO could induce not only inflammation but also the immune response. Thus, the inhibitory action of DTT on NO production has been evaluated by the study on LPS-activated mouse peritoneal macrophage. Consequently, the results implied that DTT can significantly affect cellular immunity by increasing interleukin-12 expression, Th1 response-mediated cytokine IL-2, and decreasing Th2 response-mediated cytokines IL-4 and IL-6 expression through suppressing NO production.

### 4.4. Anti-Diabetic Activity

As early as 2010, the inhibitory effect of the DTT 3β-acetoxy-20-oxo-21-nordammaran-23-oic acid (**78**) on diabetes through α-glucosidase suppressing activity has been speculated [60]. Since then, in vitro assays towards the protein tyrosine phosphatase 1B (PTP1B) were developed to evaluate their bioactivity against diabetes. As a result, five DTT with furan ring in their side-chain, gypensapogenins E (**79**), F (**80**), and G (**81**); (20*S*,23*S*)-3β,20-dihydroxyldammarane-24-ene-21-oic acid-21,23-lactone (**82**); and (20*R*,23*R*)-3β,20-dihydroxyldammarane-24-ene-21-oic acid-21,23-lactone (**83**) [160], were found to have the inhibitory ability towards the enzyme activity of PTP1B. Among them, the inhibitory activity of **79** and **81** are stronger than **80**, which suggest that the activity of DTT might be associated with the -OH in C-3 and the configuration in C-23 of the aglycone; on the other hand, the configuration of C-20 and -23 played important role to inhibitory activity of PTP1B (**82** vs. **83**) (Figure 9).

### 4.5. Other Biological Activities

**Figure 10:**
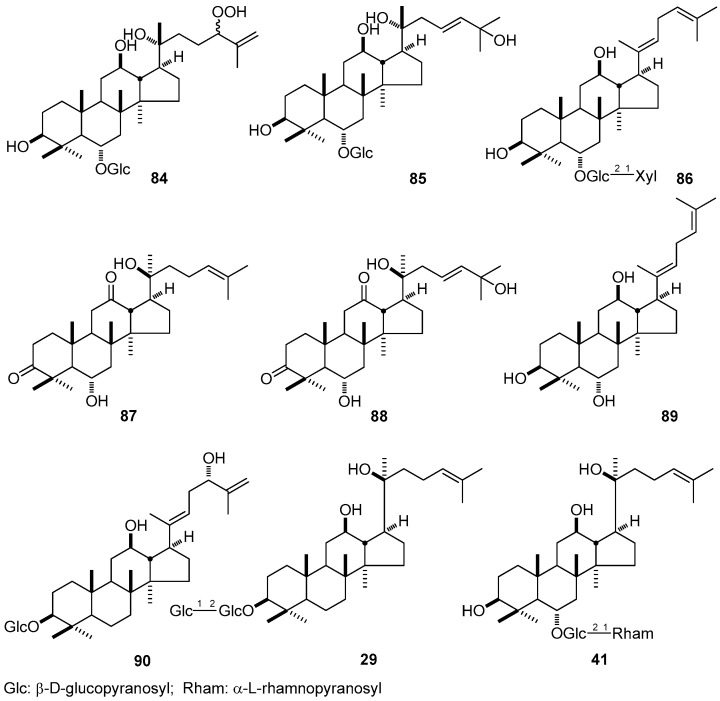
The structure of compounds **29**, **41**, **84**–**90.**

PC12 cells were used to evaluate their neurite outgrowth promoting effects of DTT. Consequently, 20*R*-ginsenoside SL_1_ (**84**), 20*R*-ginsenoside ST_2_ (**85**), and 3β,12β-dihydroxydammarane-(*E*)-20(22),24-diene-6-*O*-β-d-xylopyranosyl(1→2)-β-d-glucopyranoside (**86**) obtained from *P. notoginseng*, decreased the ratio of the neurite-bearing cells percentage, and exhibited moderate enhancing activity of the neurite outgrowth of NGF-mediated PC12 cells [161] (Figure 10).

On the other hand, as the bioactive constituents in *P. ginseng*, 6α,20*S*-dihydroxydammar-3,12-dione-24-ene (**87**) [162], 6α,20*S*,25-trihydroxydammar-3,12-dione-23-ene (**88**) [162], dammar-20(22)*E*,24-diene-3β,6α,12β-triol (**89**) [11,162], 20*S*-ginsenoside Rg_3_ (**29**) [136], ginsenoslaloside-I (**90**) [11], and 20*S*-ginsenoside Rg_2_ (**41**) [136,163] (Figure 10) showed silent information regulator two homolog 1 (SIRT1) activation activity.

Generally, based on the above-mentioned studies, numerous investigations suggested that the kinds of activities of different DTT would be related to the types of aglycone and glycoside and the number of sugars linked to the dammarane skeleton. This information may be useful for evaluating the SARs of other dammarane-type sapogenins and for developing novel antineoplastic agents.

## 5. Conclusions

As an important secondary metabolite from numerous herbal medicines, DTT have generated a great amount of interest in the field of new drug research and development. This paper summarized plant resources, NMR spectral characteristic and pharmacological function of DTT on the basis of literatures published over the last few decades.

In the field of plant resources and NMR spectral characteristic, DTT from 46 families have been summarized. Although the planar structures of DTT have been elucidated more and more clearly by 1D and/or 2D NMR and other spectroscopes, the absolute configuration still cannot be identified comprehensively. The more precise explanation of the change of chemical shift caused by diversity substitutions should be established.

In the field of pharmacological activities, natural DTT showed various activities, including anti-cancer, anti-inflammation, immunodeficiency, anti-diabetes, and so on. Especially, SARs were deeply investigated in several kinds of tumor cell lines and animal implanted with sarcoma model, which can be utilized in future as lead compounds discovery. However, the anti-tumor mechanism and in vivo research are not enough, which restrict further application in drug development.

## Figures and Tables

**Table 1:** Plant sources of DTT.

No.	Family	Genus	Species	References
1	Anacardiaceae	Mangifera	*M. indica*	[4]
		Pistacia	*P. terebinthus*	[5]
		Rhus	*R. chinensis*	[6]
2	Apocynaceae	Nerium	*N. oleander*	[7]
		Plumeria	*P. obtuse*	[8]
3	Araliaceae	Hedera	*H. rhombea*	[9]
		Panax	*P. japonicas*	[10]
			*P. ginseng*	[11]
			*P. notoginseng*	[12]
			*P. quinquefolium*	[13]
			*P. vietnamensis*	[14]
			*P. vietnamensis var. fuscidiscus*	[14]
		Polyscias	*P. fulva*	[15]
		Schefflera	*S. arboricola*	[16]
			*S. heptaphylla*	[17]
4	Betulaceae	Alnus	*A. nepalensis*	[18]
			*A. serrulatoides*	[19]
		Betula	*B. maximowicziana*	[20]
			*B. pendula*	[21]
			*B. platyphylla var. japonica*	[22]
			*B. ovalifolia*	[23]
5	Boraginaceae	Cordia	*C. multispicata*	[24]
			*C. spinescens*	[25]
			*C. verbenacea*	[26]
6	Burseraceae	Boswellia	*B. freerana*	[27]
		Commiphora	*C. confusa*	[28]
			*C. dalzielii*	[29]
			*C. incise*	[30]
			*C. kua*	[31]
			*C. myrrha*	[32]
7	Capparaceae	Cleome	*C. Africana*	[33]
			*C. amblyocarpa*	[34]
			*C. brachycarpa*	[35]
			*C. gynandra*	[36]
8	Caprifoliaceae	Viburnum	*V. cylindricum*	[37]
			*V. dilatatum*	[38]
9	Celastraceae	Celastrus	*C. rosthornianus*	[39]
		Elaeodendron	*E. buchananii*	[40]
		Maytenus	*M. macrocarpa*	[41]
10	Combretaceae	Combretum	*C. inflatum*	[42]
			*C. nigricans*	[43]
11	Commelinaceae	Commelina	*C. undulate*	[44]
12	Compositae	Arnica	*A. lonchophylla*	[45]
		Kalimeris	*K. indica*	[46]
		Saussurea	*S. oligantha*	[47]
13	Convolvulaceae	Operculina	*O. turpethum*	[48]
14	Cucurbitaceae	Actinostemma	*A. lobatum*	[49]
		Gynostemma	*G. pentaphyllum*	[50]
			*G. cardiospermum*	[51]
			*G. compressum*	[52]
			*G. yixingense*	[53]
		Luffa	*L. operculata*	[54]
		Momordica	*M. charantia*	[12]
		Neoalsomitra	*N. integrifoliola*	[55]
15	Cyperaceae	Cyperus	*C. rotundus*	[56]
16	Davidiaceae	Davidia	*D. involucrata*	[57]
17	Ericaceae	Gaultheria	*G. yunnanensi*	[58]
18	Euphorbiaceae	Homonoia	*H. riparia*	[59]
19	Fagaceae	Castanea	*C. mollissima*	[60]
20	Flacourtiaceae	Oncoba	*O. manii*	[61]
21	Gentianaceae	Gentiana	*G. rigescens*	[62]
22	Hippocrateaceae	Salacia	*S. chinensis*	[63]
23	Juglandaceae	Cyclocarya	*C. paliurus*	[64]
24	Labiatae	Glechoma	*G. longituba*	[65]
		Salvia	*S. aspera*	[66]
			*S. barrelieri*	[67]
			*S. hierosolymitana*	[68]
		Phlomis	*P. umbrosa*	[69]
25	Lauraceae	Machilus	*M. yaoshansis*	[70]
26	Leguminosae	Astragalus	*A. membranaceus*	[12]
		Erythrophleum	*E. fordii*	[71]
27	Martyniaceae	Ibicella	*I. lutea*	[72]
		Probosidea	*P. Louisiana*	[72]
28	Meliaceae	Aglaia	*A. elliptica*	[73]
			*A. erythrosperma*	[74]
			*A. eximia*	[75]
			*A. forbesii*	[76]
			*A. foveolata*	[77]
			*A. lawii*	[78]
			*A. odorata*	[79]
			*A. silvestris*	[80]
			*A. smithii*	[81]
			*A. tomentosa*	[78]
		Amoora	*A. yunnanensis*	[82]
		Chisocheton	*C. cumingianus*	[83]
			*C. penduliflorus*	[84]
			*C. polyandrous*	[85]
		Dysoxylum	*D. binectariferum*	[86]
			*D. cauliflorum*	[87]
			*D. densiflorum*	[88]
			*D. hainanense*	[89]
			*D. hongkongense*	[90]
			*D. malabaricum*	[91]
			*D. mollissimum*	[92]
			*D. muelleri*	[93]
			*D. richii*	[94]
		Walsura	*W. chrysogyne*	[95]
29	Moraceae	Ficus	*F. pumila*	[96]
30	Myricaceae	Myrica	*M. rubra*	[97]
31	Myrsinaceae	Rapanea	*R. umbellate*	[98]
			*R. lancifolia*	[98]
			*R. guyanensis*	[98]
32	Oleaceae	Forsythia	*F. suspense*	[99]
			*F. koreana*	[100]
		Ligustrum	*L. lucidum*	[101]
33	Palmae	Borassus	*B. flabellifer*	[102]
34	Polypodiaceae	Pyrrosia	*P. lingua*	[103]
		Polypodiodes	*P. niponica*	[104]
35	Pterobryaceae	Esenbeckia	*E. yaxhoob*	[105]
36	Rhamnaceae	Colubrina	*C. elliptica*	[106]
		Gouania	*G. lupuloides*	[107]
		Hovenia	*H. acerba*	[108]
			*H. dulcis*	[109]
		Zizyphus	*Z. glabrata*	[110]
			*Z. joazeiro*	[111]
			*Z. jujuba*	[112]
			*Z. lotus*	[113]
			*Z. spinosi*	[114]
			*Z. xylopyra*	[115]
37	Rhizophoraceae	Bruguiera	*B. gymnorrhiza*	[116]
		Ceriops	*C. tagal*	[117]
38	Rhoipteleaceae	Rhoiptelea	*R. chiliantha*	[118]
39	Rosaceae	Cerasus	*C. yedoensis*	[119]
40	Rubiaceae	Gardenia	*G. aubryi*	[120]
			*G. collinsae*	[121]
			*G. urvillei*	[122]
41	Sapindaceae	Eurycorymbus	*E. cavaleriei*	[123]
		Sapindus	*S. mukorossi*	[124]
42	Scrophulariaceae	Bacopa	*B. monnieri*	[125]
43	Simaroubaceae	Ailanthus	*A. altissim*	[126]
			*A. excelsa*	[127]
		Brucea	*B. javanica*	[128]
44	Sinopteridaceae	Notholaena	*N. greggii*	[129]
			*N. rigida*	[130]
45	Tiliaceae	Corchorus	*C. capsularis*	[131]
46	Umbelliferae	Centella	*C. asiatica*	[132]

**Table 2:** ^13^C-NMR data for the aglycone parts of compounds **1**–**33**.

No.	1 ^a^ [133]	1 ^b^ [134]	2 ^a^ [133]	2 ^b^ [134]	3 ^b^ [135]	4 ^a^ [59]	5 ^a^ [136]	6 ^a^ [136]	7 ^b^ [72]	8 ^b^ [134]	9 ^b^ [134]	10 ^b^ [72]	11 ^a^ [137]	12 ^a^ [138]	13 ^b^ [119]	14 ^a^ [59]	15 ^a^ [135]	16 ^b^ [139]	17 ^a^ [59]	18 ^a^ [140]	19 ^a^ [140]	20 ^a^ [55]	21 ^b^ [141]	22 ^b^ [142]	23 ^a^ [143]	24 ^a^ [143]	25 ^a^ [144]	26 ^a^ [144]	27 ^a^ [133]	28 ^a^ [145]	29 ^a^ [133]	30 ^a^ [145]	31 ^a^ [146]	32 ^a^ [133]	33 ^a^ [133]
**1**	39.2	39.1	39.5	39.0	39.0	39.8	40.3	40.2	33.6	33.5	39.0	76.1	39.6	41.7	39.9	40.6	39.0	38.5	39.6	34.2	34.3	33.5	39.0	38.9	39.5	39.5	39.4	39.4	39.3	39.4	39.2	39.4	38.9	39.3	39.2
**2**	28.0	26.7	28.2	27.4	27.4	28.5	28.0	27.8	25.4	25.3	27.4	35.9	25.9	28.7	34.1	28.5	28.1	27.1	28.6	26.5	26.5	25.4	27.3	27.5	28.2	28.3	28.3	28.3	26.8	27.9	26.9	28.7	26.8	26.8	26.6
**3**	78.3	78.4	77.9	78.8	78.9	78.7	79.5	79.5	76.2	76.1	78.9	76.6	78.1	78.0	218.2	78.1	77.6	78.6	78.8	75.3	75.3	76.2	78.8	78.9	78.0	77.9	78.0	78.0	88.9	78.6	89.0	78.7	88.9	88.8	88.8
**4**	40.2	39.1	39.5	39.0	39.1	40.7	40.0	40.5	37.6	37.6	39.0	37.4	39.5	40.3	47.4	38.8	39.7	38.9	40.8	38.1	38.1	37.5	37.5	38.9	39.6	39.6	40.0	40.0	39.8	40.4	39.7	40.2	39.7	39.7	39.6
**5**	61.7	61.0	56.3	56.0	55.9	62.1	57.3	62.1	49.5	49.5	55.9	48.1	54.2	56.9	55.3	66.3	54.1	55.7	62.2	49.7	49.4	49.5	55.7	55.9	56.4	56.4	56.4	56.4	56.5	61.5	56.5	61.4	56.4	56.5	56.3
**6**	67.6	68.4	18.7	18.3	18.3	67.9	18.9	69.9	18.2	18.2	18.3	18.1	27.6	18.7	19.6	212.3	37.1	18.3	67.9	18.6	18.6	18.2	18.2	18.3	18.8	18.8	18.8	18.8	18.6	80.1	18.5	79.9	18.4	18.5	18.4
**7**	47.4	46.8	35.2	34.8	35.2	48.3	35.9	47.3	34.7	35.1	34.8	34.6	74.7	36.6	34.5	53.7	214.0	33.9	48.4	35.2	35.2	34.8	35.3	34.9	35.4	35.4	35.2	35.2	35.3	45.2	35.2	45.1	35.0	35.2	35.1
**8**	41.1	40.8	40.0	39.8	40.4	41.9	40.9	42.0	39.9	40.5	39.8	40.5	46.0	41.1	40.2	47.3	56.0	40.2	40.8	40.2	40.2	40.0	40.8	39.8	40.2	40.3	37.4	37.4	40.2	41.1	40.0	41.2	39.9	40.1	40.0
**9**	50.1	49.5	50.4	50.2	50.6	50.6	50.9	50.7	49.9	45.3	50.1	50.7	50.4	56.1	50.0	51.1	51.1	53.4	50.9	50.6	50.5	49.8	50.6	49.9	51.0	50.9	50.6	50.5	49.3	50.2	50.4	50.1	50.2	50.3	50.1
**10**	39.3	39.2	37.3	37.1	37.1	39.7	38.2	40.2	37.2	36.9	37.1	43.3	37.6	39.6	36.8	44.5	37.2	37.5	39.9	37.7	37.7	37.3	37.1	37.1	37.5	37.5	39.6	39.6	37.1	39.7	37.0	39.7	37.0	37.0	36.9
**11**	31.9	30.9	32.0	31.2	21.5	22.0	32.0	32.0	30.9	29.0	31.2	34.3	32.3	70.5	22.0	22.6	31.2	39.1	22.0	31.7	32.5	30.4	21.2	30.6	32.6	32.3	32.6	32.3	32.3	32.1	32.0	32.1	27.9	30.9	30.6
**12**	70.9	70.5	70.9	70.8	25.4	27.7	71.9	71.9	71.0	68.4	70.7	72.1	71.1	40.8	27.5	27.4	70.8	214.1	28.0	71.1	70.7	70.6	24.7	69.9	72.5	72.6	71.0	71.0	70.9	71.0	71.0	71.0	78.5	70.2	70.1
**13**	48.1	47.2	48.5	47.7	42.3	41.0	49.5	49.6	47.7	45.3	48.5	46.7	48.7	41.1	42.3	41.3	49.9	56.2	41.2	48.4	49.4	48.9	46.7	49.2	52.4	50.4	48.6	48.6	48.7	48.3	48.6	48.3	46.8	49.5	49.4
**14**	51.5	51.3	51.6	51.6	50.3	48.3	52.6	52.5	51.7	48.8	51.6	51.2	52.0	50.6	50.2	48.4	50.2	54.7	45.8	52.2	52.3	51.7	49.8	51.2	51.2	51.0	51.8	51.7	51.9	51.7	51.8	51.7	52.2	51.5	51.3
**15**	31.3	30.9	31.8	31.1	31.2	44.6	32.0	31.4	31.1	31.3	31.1	30.9	36.1	31.3	31.1	44.5	33.2	30.8	50.5	32.3	32.2	31.3	31.2	31.1	33.8	32.6	31.5	31.4	31.5	31.4	31.4	31.2	31.3	30.8	30.6
**16**	26.8	25.4	26.8	25.5	27.6	74.2	27.1	27.1	26.5	24.1	26.4	26.1	28.5	25.5	24.8	73.9	27.0	24.7	220.6	25.5	25.7	26.8	28.7	25.2	30.8	28.7	27.0	26.9	26.8	26.8	26.8	26.8	27.2	26.7	26.6
**17**	54.6	53.5	54.7	53.6	49.9	52.2	51.3	50.9	53.5	46.9	49.9	54.4	54.2	50.3	49.8	52.5	53.8	46.1	58.5	49.9	49.9	50.6	37.5	54.7	48.2	50.9	54.6	54.8	54.8	54.8	54.8	54.8	54.1	51.7	51.5
**18**	17.5	17.2	16.2	16.2	16.2	17.8	16.3	17.6	16.5	15.9	16.2	15.9	10.7	17.0	15.2	16.7	15.3	15.9	18.0	16.5	16.6	15.5	15.5	15.6	15.8	15.9	16.5	16.5	16.5	17.4	15.9	17.4	16.3	16.3	16.2
**19**	17.4	17.2	15.8	15.7	15.5	18.8	16.8	17.7	15.7	15.2	15.7	11.7	16.7	17.0	16.0	17.6	15.9	15.8	17.9	15.6	15.7	16.0	15.3	16.1	16.3	16.5	16.3	16.3	16.0	17.7	16.4	17.6	15.7	16.0	16.0
**20**	73.9	73.9	72.9	74.0	75.4	75.1	74.7	74.7	74.4	75.3	74.5	73.4	73.3	73.9	75.4	75.1	73.3	73.1	74.6	86.7	87.1	86.4	140.3	76.7	155.5	140.1	73.0	73.1	73.0	73.0	73.0	73.0	73.0	83.4	83.3
**21**	26.9	26.7	26.9	26.8	24.8	27.0	22.4	22.4	27.0	26.9	21.8	26.2	27.3	25.8	25.4	27.1	27.1	26.4	27.0	26.9	26.9	21.3	173.9	19.4	108.1	13.2	27.5	27.4	27.2	27.1	27.1	27.0	26.9	22.5	22.3
**22**	35.7	34.5	35.8	34.8	40.5	44.3	44.0	44.0	34.7	36.3	42.3	35.3	35.2	41.8	40.4	44.2	35.6	37.8	41.3	32.8	32.5	39.1	145.7	35.8	32.7	123.6	32.1	32.1	36.0	35.8	35.9	35.8	36.5	36.2	36.1
**23**	22.9	22.3	22.9	22.4	22.6	23.6	19.4	18.9	22.4	22.4	21.8	22.4	23.2	23.3	22.5	23.6	23.0	22.4	23.6	28.7	28.6	25.9	78.0	16.3	27.1	27.4	30.8	30.6	23.1	23.0	23.0	23.0	23.0	23.3	23.1
**24**	126.2	125.0	126.2	125.2	124.7	126.4	45.4	45.4	125.0	124.7	124.6	125.3	126.5	126.0	124.6	126.4	126.4	124.9	125.9	85.6	88.3	86.4	121.9	36.5	125.3	123.9	76.4	76.0	126.2	126.4	126.4	126.3	126.6	126.0	126.0
**25**	130.5	131.4	130.6	131.4	131.6	131.1	71.5	71.5	131.8	131.9	131.9	131.2	130.8	130.7	131.7	131.2	131.0	131.5	131.5	70.1	70.0	70.2	137.8	73.1	131.2	131.2	150.2	150.0	130.0	130.8	130.8	130.8	130.9	131.0	130.8
**26**	25.8	25.7	25.8	25.8	25.7	26.2	29.4	29.1	25.7	25.7	25.8	25.8	25.9	26.1	25.7	26.2	26.0	25.7	26.2	27.3	26.5	24.6	25.7	33.0	25.8	25.7	110.1	109.8	25.9	25.8	25.9	25.8	25.8	25.8	25.7
**27**	17.7	17.7	17.6	17.8	17.7	18.1	29.1	29.4	17.7	17.7	17.8	17.7	17.8	17.7	17.7	18.1	17.8	17.7	18.1	27.6	29.0	28.0	18.2	27.1	17.7	17.7	18.2	18.4	17.5	17.7	17.7	17.7	17.7	17.8	17.8
**28**	31.9	30.9	28.6	28.1	28.0	32.4	28.6	31.9	28.3	28.3	28.1	28.0	28.8	29.0	26.7	28.5	28.0	28.0	32.4	29.4	29.4	28.3	27.9	28.0	28.7	28.8	28.7	28.7	28.2	31.7	28.2	32.1	28.1	28.2	28.0
**29**	16.4	15.5	16.4	15.5	15.4	16.9	16.2	16.1	22.1	22.2	15.4	22.1	16.6	16.6	21.0	16.6	15.6	15.3	16.9	22.4	22.4	22.1	15.6	15.3	16.6	16.3	15.8	15.9	16.9	16.4	16.6	16.8	16.6	16.8	16.7
**30**	17.0	16.9	17.0	16.9	16.5	18.2	17.4	17.4	17.0	19.4	17.2	16.5	17.2	16.8	16.3	18.9	18.8	17.5	17.5	18.2	18.0	17.0	16.1	17.1	17.0	17.0	17.1	17.1	16.9	16.8	17.1	16.8	17.4	17.4	17.5

^a^ determined in C_5_D_5_N; ^b^ determined in CDCl_3_.
